# Chromosome-level genome assembly of the *Drosicha contrahens*

**DOI:** 10.1038/s41597-026-07406-w

**Published:** 2026-05-18

**Authors:** Yu-ang Li, Xinyi Zheng, Han Xu, San-an Wu

**Affiliations:** The Key Laboratory for Silviculture and Conservation of Ministry of Education, Beijing Forestry University, Beijing, 100083 P. R. China

**Keywords:** Genome, Taxonomy

## Abstract

*Drosicha contrahens* is an important forestry pest in China, primarily infesting fruit trees. In this study, we assembled a chromosome-level genome of *D. contrahens* using BGI short reads, PacBio Revio long reads, and Hi-C sequencing data. The assembled genome size is 681.68 Mb, with a scaffold N50 of 282.16 Mb. Of this, 677.81 Mb (99.43%) were successfully anchored onto 3 chromosomes. In the genome annotation, a total length of 382.47 Mb of repetitive sequences were identified, along with 12,174 predicted protein-coding genes and 5,644 non-coding RNAs. This high-quality genome assembly of *D. contrahens* provides a comprehensive molecular resource for scale insects, which will aid in the development of novel pest control strategies in the future. Additionally, it contributes to advancing understanding of the phylogeny and evolution of scale insects.

## Background & Summary

Scale insects (Coccomorpha), obligate plant-parasitic insects morphologically mimicking host plant structures, are distributed in all zoogeographic regions except Antarctica^1,2^. The classification of Coccomorpha primarily relies on morphological characteristics of adult females, as males are small and have short life cycles, making field collection very difficult. However, adult females exhibit neotenic features with highly reduced morphological characteristics, leading to persistent controversies over higher-level phylogenetic relationships within scale insects^2–4^. Additionally, genome assembly in scale insects faces significant technical challenges, including contamination from endosymbionts, abundant repetitive sequences, and high heterozygosity. Consequently, phylogenetic studies within Coccomorpha based on molecular markers remain limited.

**Fig. 1:**
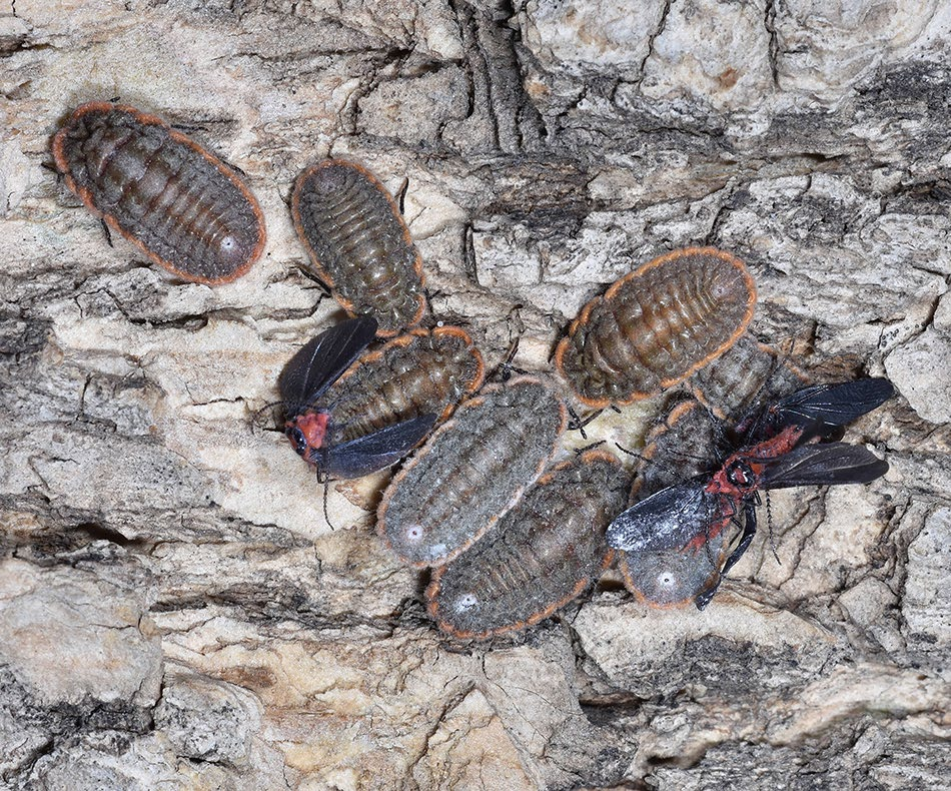
Ecological photo of *D*. *contrahens*.

The family Monophlebidae comprises 267 recognized species across 49 genera. Within this family, the genus *Drosicha* has 25 species distributed worldwide, with *Drosicha contrahens* designated as the type species^5^. Notably, four species of this genus are documented agricultural pests (*D. corpulenta*, *D. mangiferae*, *D. stebbingii*, and *D. townsendi*)^6^. However, *D. contrahens* (Fig. 1) is widely distributed in China and poses significant threats to fruit-bearing trees spanning multiple botanical families, including Anacardiaceae, Lauraceae, Moraceae, Phyllanthaceae, Rosaceae and Rutaceae, with extremely high population densities during its infestation period. Furthermore, this species demonstrates explosive population growth during infestation periods^7,8^. Consequently, *D*. *contrahens* is a potential economic forest pest.

In this study, we generated a chromosome-scale reference genome assemblyfor *D. contrahens* through integrated PacBio long-read sequencing and high-throughput chromosome conformation capture (Hi-C) scaffolding. The final assembly has a total size of 681.68 Mb with scaffold N50 of 282.16 Mb, of which 677.81 Mb (99.43%) were successfully anchored onto three chromosomes. The assembly was subsequently annotated for repetitive sequences, protein-coding genes (with functional annotation), and non-coding RNAs. Our chromosome-level genome assembly not only enriched the genomic database of Coccomorpha but also provided a valuable resource for future phylogenetic studies and insights into the origin and diversification of scale insects. Furthermore, the identification of gene families provides actionable insights for developing targeted techniques against scale insects as forestry pest.

## Methods

### Sample preparation and sequencing

Adult females of *Drosicha contrahens* was collected on *Fraxinus pennsylvanica* on May 5, 2023, at Beijing Forestry University, Beijing city, China. Fresh samples had the abdominal tissue posterior to the coxae of the hind legs removed to eliminate the influence of endosymbionts, and were then promptly flash-frozen in liquid nitrogen and stored at −80 °C for future use.

DNA extraction was performed using column-based method. Subsequently, three libraries were constructed: Genome survey library, PacBio library and Hi-C library. For the Genome survey, 10 adult females were used. Library preparation was performed using the BGI Optimal DNA Library Prep Kit (BGI-Shenzhen, China), and sequencing reads of 150 bp were generated on the DNBSEQ platform (BGI-Shenzhen, China). The PacBio library was constructed using 10 adult females with the SMRTbell Template Prep Kit, and HiFi sequencing was performed on the PacBio Revio platform. Finally, the Hi-C library was constructed using 10 samples and sequenced on the DNBSEQ platform through combinatorial probe-anchor synthesis (cPAS) to assemble a chromosome-level genome.

For transcriptome sequencing, RNA was extracted from 5 fresh adult females using the TRIzol method (Invitrogen, Carlsbad, California, USA)^9^ following the manufacturer. Library preparation was performed using the Optimal Dual-mode mRNA Library Prep Kit (BGI-Shenzhen, China), and sequencing was conducted on the MGISEQ-2000 platform. This generated approximately 10 Gb of raw data per library.

### Genome assembly

The raw data were first filtered using fastp v0.20.0^10^ (--average_qual 15 -l 150) to remove adapters and low-quality sequences. Subsequently, K-mer frequencies (K = 17) were counted using Jellyfish v2.2.6^11^, and the genome size and heterozygosity were estimated by GenomeScope^12^ based on the K-mer frequency distribution. The final preliminary estimated genome size is 729.36 Mb, with a heterozygosity rate of 1.19% and a duplication rate of 64.6%.

**Fig. 2:**
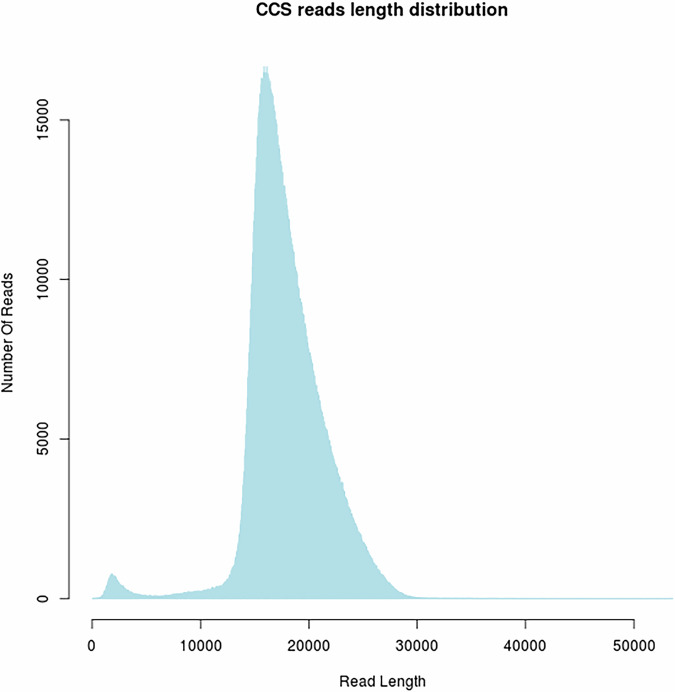
Circular Consensus Sequencing (CCS) read length distribution.

The contig-level genome assembly was performed using approximately 110.25 Gb of PacBio HiFi reads (see read length distribution in Fig. 2) with hifiasm^13^,followed by the removal of redundant heterozygous contigs using purge_haplotigs^14^ to mitigate the effects of high heterozygosity introduced by sample pooling. The assembled genome was reported to have a total length of 928.68 Mb, comprising 1494 contigs, with an N50 of 814,855 bp, a maximum contig length of 2,680,917 bp, and a GC content of 31.93%. The initial hifiasm assembly spanned 928.68 Mb, which was larger than both the k-mer-based estimate and the final assembly size due to the retention of redundant sequences caused by high heterozygosity. The assembly demonstrated high base-level accuracy, with a consensus quality value (QV) of 51.39 as assessed by Merqury v1.3^15^ based on long-read data.

**Fig. 3:**
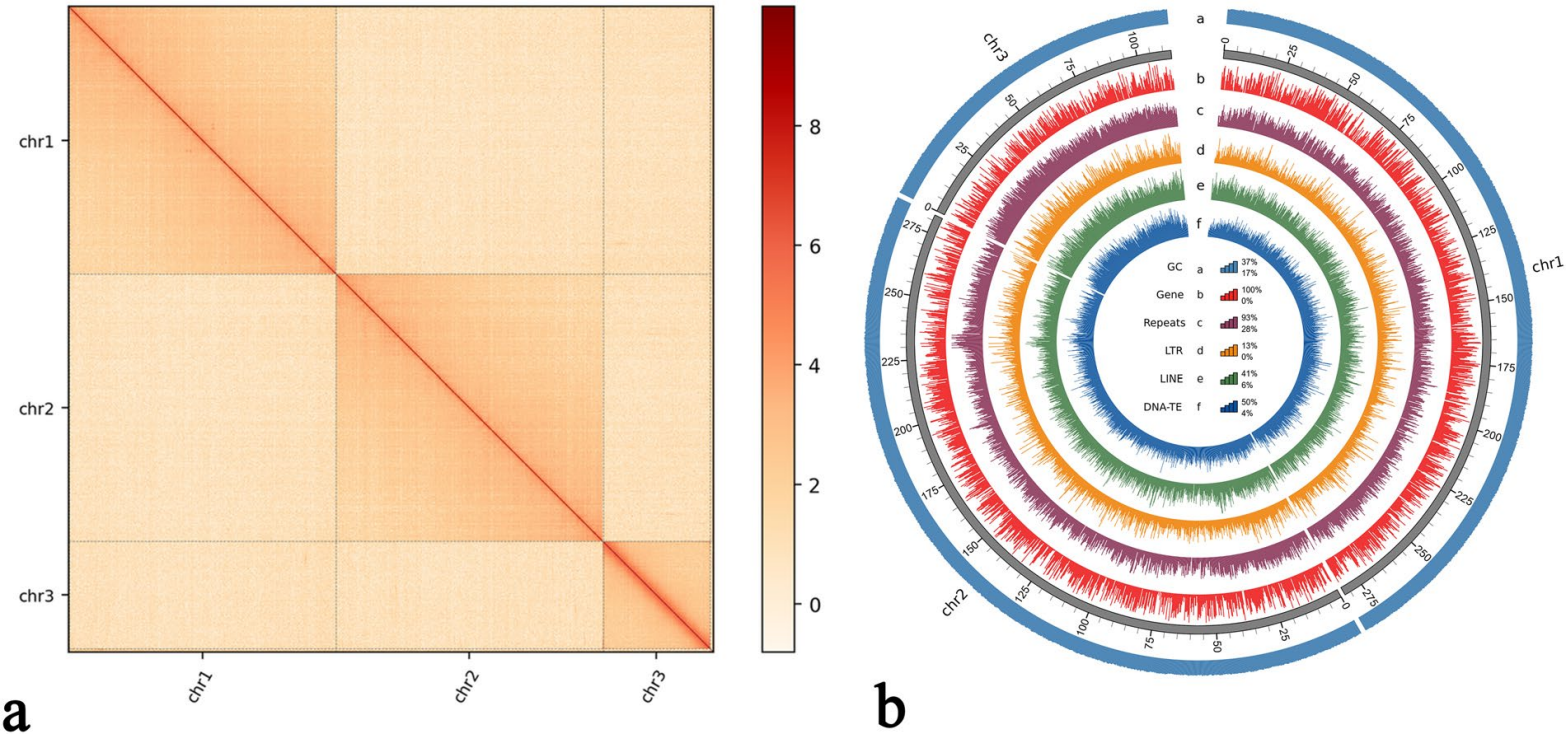
Genomic heatmap and features. **(a**) Heatmap of genome-wide Hi-C interaction within 3 chromosomes. (**b**) Genomic landscape of the 3 chromosomes, with a window size of 300 kb. Each circle from outside (a) to inside (f) represents GC content, gene density, total repeat sequence density distribution, LTR density distribution, LINE density, and DNA-TE density distribution.

The Hi-C data were subjected to quality control using fastp v0.20.0 (-l 50), and the quality of the processed data was evaluated using HiCUP v0.7.2^16^. The assembled genome was aligned and filtered using Juicer v1.5.6^17^, and then chromosome scaffolding was performed using 3D-DNA v180922^18^ (-r 0), followed by manual curation of the Hi-C contact map in Juicebox v1.11.08^19^ through visual inspection to correct misassemblies in contig order, orientation, and internal joins. During Hi-C scaffolding, redundant sequences were identified and removed, resulting in a final chromosome-level genome assembly of 681.68 Mb (including 58 scaffolds, with N50 size of 282.16 Mb). Hi-C scaffolding successfully anchored 677.81 Mb (99.43%) of the genome to three chromosomes (Fig. 3). The three pseudo-chromosomes were directly supported by the Hi-C contact map, which showed three distinct diagonal blocks of strong intra-chromosomal interactions (Fig. 3a). Their lengths and gene contents are summarized in Table 1. The BUSCO v5.7.1^20^ (--miniprot -m genome -f --offline) analysis (insecta_odb10, 2024-1-8) indicated 86.4% genome completeness, with 83.5% single-copy and 2.9% duplicated BUSCOs.

**Table 1:** Statistics of the three assembled chromosomes.

Chromosome	Length (Mb)	Number of anchored contigs
Chr1	282.46	772
Chr2	282.16	771
Chr3	113.18	330

### Genome annotation

The annotation of repetitive sequences was performed using the following three methods: 1) *De novo* assembly based on repetitive sequence features using TRF v4.09^21^ (2 7 7 80 10 50 2000 -d -h). 2) Homology-based prediction using RepeatMasker open-4.0.9 (http://www.repeatmasker.org) (-nolow -no_is -norna) and its internal module RepeatProteinMask based on the RepBase database. 3) Repetitive sequence families were identified through *de novo* prediction using RepeatModeler open-1.0.11^22^, and long terminal repeat retrotransposons (LTR-RTs) were detected using LTR_FINDER_parallel v1.07^23^, leading to the construction of a library. RepeatMasker was used to annotate repetitive sequences in the genome based on this library. Finally, the results from all three methods were integratedand merged using BEDTools v2.18^24^ to remove redundant overlapping regions, resulting in a total sequence size of 382.47 Mb (56.11% of the genome) (Table 2).

**Table 2:** Summary of repetitive sequences identified in the genome of *D*. *contrahens* using different methods.

Type	Repeat Size	% of genome
Trf	35344674	5.18
Repeatmasker	127116684	18.65
Proteinmask	89647543	13.15
*De novo*	311276930	45.66
Total	382472004	56.11

For the prediction of protein-coding genes, we implemented a multi-strategy approach to ensure comprehensive annotation. Initially, ab initio prediction was performed using both Genscan^25^ and AUGUSTUS v3.3.2^26^. Subsequently, for homology-based prediction, five closely species, including *Aphis gossypii*, *Kerria lacca*, *Parthenolecanium corni*, *Planococcus citri* and *Rhopalosiphum padi* (Table 3), were selected for homology-based prediction, with Miniprot v0.11 (r234)^27^ (-gff-only -O 11 -E 1 -F 23 -C 1 -B 5 -G 200000 -j 1) used for sequence alignment. For enhanced prediction accuracy, Liftoff v1.6.3^28^ (-a 0.5 -s 0.5) was employed to predict protein sequences from homologous species. Regarding transcript-based approaches, RNA sequencing data were first aligned to the reference genome through HISAT2 v2.1.0^29^ (--dta), followed by transcript assembly using StringTie v1.3.5^30^. The resulting transcripts were then subjected to gene prediction via TransDecoder v5.5.0 (Haas, BJ. https://github.com/TransDecoder/TransDecoder). To integrate these data, MAKER2 v2.31.10^31^ pipeline (max_dna_len = 3000000 min_contig = 10000 pred_flank = 500 min_protein = 30) was systematically applied to consolidate results from *de novo* prediction, homologous proteins comparison and transcriptome evidence. Finally, rigorous manual curation was performed to enhance the accuracy and reliability of the gene set.

**Table 3:** Species used for Homology-Based prediction.

Family	Species	Version
Kerriidae	*Kerria lacca*	GCA_045014175.1
Coccoidae	*Parthenolecanium corni*	GCA_038050395.1
Pseudococcidae	*Planococcus citri*	GCF_950023065.1
Aphididae	*Aphis gossypii*	GCA_917880025.4
Aphididae	*Rhopalosiphum padi*	GCF_020882245.1

Functional annotation of proteins within the gene set was achieved through two complementary strategies: sequence similarity analysis and domain/motif architecture characterization. To deduce functional information, protein sequences were initially aligned against the KOG/COG (2003-3-1/2020-11-25)^32^, GO (2023-4-1)^33^, KEGG (105.0, 2023-1-1)^34^, NR (2023-4-1)^35^, SwissProt (2023-3-1)^36^, and TrEMBL (2023-3-1)^36^ databases using diamond v2.0.14^37^ (--evalue 1e-05). Subsequently, InterProScan v5.61-93.0^38^ (--seqtype p --formats TSV --goterms --pathways -dp) was implemented to identify conserved domains, motifs, and functional sites through InterPro^39^ sub-database, while hmmscan (in HMMER3 V3.3.1^40^) (hmmsearch -E 1e-05 --domE 1e-05) was employed to perform high-precision annotation of transcription factors and specific domains based on hidden Markov models (HMMs).

In the annotation of non-coding RNAs, different methods were used based on the specific characteristics of each RNA type. Leveraging the structural features of tRNAs (transfer RNAs), tRNAscan-SE v1.3.1^41^ was used to identify tRNA sequences in the genome. For rRNA annotation, BLASTN v2.11.0 + ^42^ (-evalue 0.01) was used to align the genome sequence with rRNA (Ribosomal RNAs) sequences from close species. Additionally, the Rfam v14.8^43^ (cmscan --rfam --nohmmonly) was used to identify miRNA (Micro RNAs) and snRNA (Small Nuclear RNAs).

## Data Records

The raw sequencing data and genome assembly of *D*. *contrahens* have been submitted to the National Center for Biotechnology Information (NCBI) under BioProject PRJNA1237767. The PacBio, BGI, Hi-C and transcriptomic data are accessible via the accession numbers SRP571666^44^. The final chromosome assembly has been deposited in GenBank under the accession number JBMFZV000000000^45^.

## Technical Validation

We aligned the short-read data to the assembled genome using BWA v0.7.12-r1039^46^, achieving a mapping rate of 94.59%, an average sequencing depth of 76.64x, and a coverage rate of 99.94%. Alignment of the long-read data to the genome using Minimap2 v2.24-r1122^47^ yielded a mapping rate of 99.13%, an average sequencing depth of 33.48x, and a coverage rate of 99.98%.

The completeness of the genome assembly was further assessed using BUSCO v5.7.1 with the insecta_odb10 database, recovering 86.4% of complete BUSCOs (83.5% single-copy, 2.9% duplicated). This level of completeness is comparable to other scale insect genomes and is largely attributed to the high repeat content and heterozygosity of *D. contrahens*.

## Data Availability

The Illumina, PacBio, and Hi-C sequencing data used for genome assembly, as well as the transcriptome data used for genome annotation, have all been deposited in the NCBI Sequence Read Archive (SRA) under accession numbers SRR32776411–SRR32776426, with the associated BioProject accession number PRJNA1237767. Additionally, the chromosome-level genome assembly and its GFF file can be accessed via GenBank under accession number GCA_054252755.1.

## Code availability

In this study, no custom scripts or code were used for processing and validating the dataset. All software parameters are mentioned in the Methods section, and any unspecified parameters were set to their default values.

## References

[1] Cook, L. G., Gullan, P. J. & Trueman, H. E. A preliminary phylogeny of the scale insects (Hemiptera: Sternorrhyncha: Coccoidea) based on nuclear small-subunit ribosomal DNA. *Mol. Phylogenet. Evol.* **25**, 43–52, 10.1016/s1055-7903(02)00248-8 (2002). 12383749 10.1016/s1055-7903(02)00248-8

[2] Gullan, P. J. & Cook, L. G. Phylogeny and higher classification of the scale insects (Hemiptera: Sternorrhyncha: Coccoidea). *Zootaxa*, 413-425 (2007).

[3] Hardy, N. B. The status and future of scale insect (Coccoidea) systematics. *Syst. Entomol.* **38**, 453–458, 10.1111/syen.12022 (2013).

[4] Hodgson, C. A Review of Neococcid Scale Insects (Hemiptera: Sternorrhyncha: Coccomorpha) Based on the Morphology of the Adult Males. *Zootaxa* **4765**, 1–264, 10.11646/zootaxa.4765.1.1 (2020). 10.11646/zootaxa.4765.1.1 33056615

[5] García Morales, M. *et al*. ScaleNet: A literature-based model of scale insect biology and systematics. *Database* **2016**, bav118, 10.1093/database/bav118 (2016). 26861659 10.1093/database/bav118 PMC4747323

[6] Kondo, T. & Watson, G. W. in *Encyclopedia of Scale Insect Pests* (eds Takumasa Kondo & Gillian W. Watson) 8-37 (CABI, 2022).

[7] Tang, F. D. & Hao, J. J. *The Margarodidae and others of China*. (Chinese Agricultural Science Technology Press, 1995).

[8] Xu, Q., Sun, X. H., Wu, D. H. & Zhang, Z. P. Biological characteristics and control methods of *Drosicha corpulenta* (Kanawa). *Journal of Jiangsu Forestry Science and Technology* **26**(1), 52–54 (1999).

[9] Rio, D. C., Ares, M. Jr., Hannon, G. J. & Nilsen, T. W. Purification of RNA using TRIzol (TRI reagent). *Cold Spring Harbor protocols* **2010**, pdb.prot5439–pdb.prot5439, 10.1101/pdb.prot5439 (2010). 20516177 10.1101/pdb.prot5439

[10] Chen, S., Zhou, Y., Chen, Y. & Gu, J. fastp: an ultra-fast all-in-one FASTQ preprocessor. *Bioinformatics* **34**, 884–890, 10.1093/bioinformatics/bty560 (2018). 30423086 10.1093/bioinformatics/bty560 PMC6129281

[11] Marcais, G. & Kingsford, C. A fast, lock-free approach for efficient parallel counting of occurrences of *k*-mers. *Bioinformatics* **27**, 764–770, 10.1093/bioinformatics/btr011 (2011). 21217122 10.1093/bioinformatics/btr011 PMC3051319

[12] Vurture, G. W. *et al*. GenomeScope: fast reference-free genome profiling from short reads. *Bioinformatics* **33**, 2202–2204, 10.1093/bioinformatics/btx153 (2017). 28369201 10.1093/bioinformatics/btx153 PMC5870704

[13] Cheng, H., Concepcion, G. T., Feng, X., Zhang, H. & Li, H. Haplotype-resolved *de novo* assembly using phased assembly graphs with hifiasm. *Nat. Methods* **18**, 170–175, 10.1038/s41592-020-01056-5 (2021). 33526886 10.1038/s41592-020-01056-5 PMC7961889

[14] Roach, M. J., Schmidt, S. A. & Borneman, A. R. Purge Haplotigs: allelic contig reassignment for third-gen diploid genome assemblies. *BMC Bioinf*. **19**, 10.1186/s12859-018-2485-7 (2018). 10.1186/s12859-018-2485-7 PMC6267036 30497373

[15] Rhie, A., Walenz, B. P., Koren, S. & Phillippy, A. M. Merqury: reference-free quality, completeness, and phasing assessment for genome assemblies. *Genome Biol*. **21**, 10.1186/s13059-020-02134-9 (2020). 10.1186/s13059-020-02134-9 PMC7488777 32928274

[16] Wingett, S. *et al*. HiCUP: pipeline for mapping and processing Hi-C data. *F1000Research* **4**, 1310–1310, 10.12688/f1000research.7334.1 (2015). 26835000 10.12688/f1000research.7334.1 PMC4706059

[17] Durand, N. C. *et al*. Juicer Provides a One-Click System for Analyzing Loop-Resolution Hi-C Experiments. *Cell Syst.* **3**, 95–98, 10.1016/j.cels.2016.07.002 (2016). 27467249 10.1016/j.cels.2016.07.002 PMC5846465

[18] Dudchenko, O. *et al*. *De novo* assembly of the *Aedes aegypti* genome using Hi-C yields chromosome-length scaffolds. *Science* **356**, 92–95, 10.1126/science.aal3327 (2017). 28336562 10.1126/science.aal3327 PMC5635820

[19] Durand, N. C. *et al*. Juicebox Provides a Visualization System for Hi-C Contact Maps with Unlimited Zoom. *Cell Syst.* **3**, 99–101, 10.1016/j.cels.2015.07.012 (2016). 27467250 10.1016/j.cels.2015.07.012 PMC5596920

[20] Manni, M., Berkeley, M. R., Seppey, M., Simao, F. A. & Zdobnov, E. M. BUSCO Update: Novel and Streamlined Workflows along with Broader and Deeper Phylogenetic Coverage for Scoring of Eukaryotic, Prokaryotic, and Viral Genomes. *Mol. Biol. Evol.* **38**, 4647–4654, 10.1093/molbev/msab199 (2021). 34320186 10.1093/molbev/msab199 PMC8476166

[21] Benson, G. Tandem repeats finder: a program to analyze DNA sequences. *Nucleic acids research* **27**, 573–580, 10.1093/nar/27.2.573 (1999). 9862982 10.1093/nar/27.2.573 PMC148217

[22] Price, A. L., Jones, N. C. & Pevzner, P. A. *De novo* identification of repeat families in large genomes. *Bioinformatics* **21**, I351–I358, 10.1093/bioinformatics/bti1018 (2005). 15961478 10.1093/bioinformatics/bti1018

[23] Ou, S. & Jiang, N. LTR_FINDER_parallel: parallelization of LTR_FINDER enabling rapid identification of long terminal repeat retrotransposons. *Mobile DNA* **10**, 10.1186/s13100-019-0193-0 (2019). 10.1186/s13100-019-0193-0 PMC6909508 31857828

[24] Quinlan, A. R. & Hall, I. M. BEDTools: a flexible suite of utilities for comparing genomic features. *Bioinformatics* **26**, 841–842, 10.1093/bioinformatics/btq033 (2010). 20110278 10.1093/bioinformatics/btq033 PMC2832824

[25] Burge, C. & Karlin, S. Prediction of complete gene structures in human genomic DNA. *J. Mol. Biol.* **268**, 78–94, 10.1006/jmbi.1997.0951 (1997). 9149143 10.1006/jmbi.1997.0951

[26] Stanke, M. *et al*. AUGUSTUS: *ab initio* prediction of alternative transcripts. *Nucleic Acids Research* **34**, W435–W439, 10.1093/nar/gkl200 (2006). 16845043 10.1093/nar/gkl200 PMC1538822

[27] Slater, G. S. & Birney, E. Automated generation of heuristics for biological sequence comparison. *BMC Bioinf*. **6**, 10.1186/1471-2105-6-31 (2005). 10.1186/1471-2105-6-31 PMC553969 15713233

[28] Shumate, A. & Salzberg, S. L. Liftoff: accurate mapping of gene annotations. *Bioinformatics* **37**, 1639–1643, 10.1093/bioinformatics/btaa1016 (2021). 33320174 10.1093/bioinformatics/btaa1016 PMC8289374

[29] Kim, D., Paggi, J. M., Park, C., Bennett, C. & Salzberg, S. L. Graph-based genome alignment and genotyping with HISAT2 and HISAT-genotype. *Nat. Biotechnology* **37**, 907–915, 10.1038/s41587-019-0201-4 (2019). 10.1038/s41587-019-0201-4 PMC7605509 31375807

[30] Pertea, M. *et al*. StringTie enables improved reconstruction of a transcriptome from RNA-seq reads. *Nat. Biotechnology* **33**, 290–295, 10.1038/nbt.3122 (2015). 10.1038/nbt.3122 PMC4643835 25690850

[31] Holt, C. & Yandell, M. MAKER2: an annotation pipeline and genome-database management tool for second-generation genome projects. *BMC Bioinf*. **12**, 10.1186/1471-2105-12-491 (2011). 10.1186/1471-2105-12-491 PMC3280279 22192575

[32] Galperin, M. Y. *et al*. COG database update: focus on microbial diversity, model organisms, and widespread pathogens. *Nucleic Acids Research* **49**, D274–D281, 10.1093/nar/gkaa1018 (2021). 33167031 10.1093/nar/gkaa1018 PMC7778934

[33] Ashburner, M. *et al*. Gene ontology: tool for the unification of biology. The Gene Ontology Consortium. *Nat. genetics* **25**, 25–29, 10.1038/75556 (2000). 10802651 10.1038/75556 PMC3037419

[34] Kanehisa, M. & Goto, S. KEGG: kyoto encyclopedia of genes and genomes. *Nucleic Acids Research* **28**, 27–30, 10.1093/nar/28.1.27 (2000). 10592173 10.1093/nar/28.1.27 PMC102409

[35] Sayers, E. W. *et al*. Database resources of the National Center for Biotechnology Information. *Nucleic Acids Research* **37**, D5–D15, 10.1093/nar/gkn741 (2009). 18940862 10.1093/nar/gkn741 PMC2686545

[36] Coudert, E. *et al*. Annotation of biologically relevant ligands in UniProtKB using ChEBI. *Bioinformatics* **39**, 10.1093/bioinformatics/btac793 (2023). 10.1093/bioinformatics/btac793 PMC9825770 36484697

[37] Buchfink, B., Reuter, K. & Drost, H.-G. Sensitive protein alignments at tree-of-life scale using DIAMOND. *Nat. Methods* **18**, 366–368, 10.1038/s41592-021-01101-x (2021). 33828273 10.1038/s41592-021-01101-x PMC8026399

[38] Jones, P. *et al*. InterProScan 5: genome-scale protein function classification. *Bioinformatics* **30**, 1236–1240, 10.1093/bioinformatics/btu031 (2014). 24451626 10.1093/bioinformatics/btu031 PMC3998142

[39] Paysan-Lafosse, T. *et al*. InterPro in 2022. *Nucleic Acids Research* **51**, D418–D427, 10.1093/nar/gkac993 (2023). 36350672 10.1093/nar/gkac993 PMC9825450

[40] Mistry, J., Finn, R. D., Eddy, S. R., Bateman, A. & Punta, M. Challenges in homology search: HMMER3 and convergent evolution of coiled-coil regions. *Nucleic Acids Research* **41**10.1093/nar/gkt263 (2013). 10.1093/nar/gkt263 PMC3695513 23598997

[41] Lowe, T. M. & Eddy, S. R. tRNAscan-SE: a program for improved detection of transfer RNA genes in genomic sequence. *Nucleic acids research* **25**, 955–964, 10.1093/nar/25.5.955 (1997). 9023104 10.1093/nar/25.5.955 PMC146525

[42] Altschul, S. F., Gish, W., Miller, W., Myers, E. W. & Lipman, D. J. Basic local alignment search tool. *J. Mol. Biol.* **215**, 403–410, 10.1016/s0022-2836(05)80360-2 (1990). 2231712 10.1016/S0022-2836(05)80360-2

[43] Griffiths-Jones, S. *et al*. Rfam: annotating non-coding RNAs in complete genomes. *Nucleic Acids Research* **33**, D121–D124, 10.1093/nar/gki081 (2005). 15608160 10.1093/nar/gki081 PMC540035

[44] *NCBI Sequence Read Archive* http://identifiers.org/ncbi/insdc.sra:SRP571666 (2025).

[45] *NCBI Assembly*, <https://identifiers.org/ncbi/insdc:JBMFZV000000000> (2025).

[46] Li, H. & Durbin, R. Fast and accurate short read alignment with Burrows-Wheeler transform. *Bioinformatics* **25**, 1754–1760, 10.1093/bioinformatics/btp324 (2009). 19451168 10.1093/bioinformatics/btp324 PMC2705234

[47] Li, H. Minimap2: pairwise alignment for nucleotide sequences. *Bioinformatics* **34**, 3094–3100, 10.1093/bioinformatics/bty191 (2018). 29750242 10.1093/bioinformatics/bty191 PMC6137996

